# The abandonment of Australians in India: an analysis of the right of entry as a security right in the age of COVID-19

**DOI:** 10.1007/s40592-022-00151-x

**Published:** 2022-02-18

**Authors:** Diego S. Silva

**Affiliations:** Sydney Health Ethics and the University of Sydney School of Public Health, Rm 133, Edward Ford Building, A27 Fisher Rd, University of Sydney, Sydney, NSW 2006 Australia

**Keywords:** COVID-19, Human rights, Pandemic, Right of entry, Security, Security rights

## Abstract

In May 2021, when the Delta variant of SARS-CoV2 was wreaking havoc in India, the Australian Federal Government banned its citizens and residents who were there from coming back to Australia for 14 days on penalty of fines or imprisonment. These measures were justified on the grounds of protecting the broader Australian public from potentially importing the Delta strain, which officials feared would then seed a local outbreak. Those Australians stranded in India, and their families and communities back home, claimed that they were abandoned by Prime Minister Scott Morrison’s government. This case—along with other barriers used as part of border control measures in the name of public health—raises the following question: is it ever morally permissible for a state to ban its citizens and residents from entering their own country during a pandemic? I conclude that it’s impermissible. I argue that persons have a right of entry that should be understood as a security right. This security right should be non-derogable because it’s a foundational good that is necessary for life-planning purposes. Moreover, it is a right that people should be able to rely upon absolutely, even during pandemics. At the very least, should someone believe that there are rare exceptions to the right of entry on public health grounds, governments have a duty—grounded in the principle of reciprocity—to support those who are temporarily denied entry. In the case of Australians stranded in India, I will argue that the Australian Federal Government failed on all accounts.

## Introduction

In this paper, I argue that some border control measures taken by the Australian Federal Government as a response to the COVID-19 pandemic breached a right to security of the person by inappropriately derogating Australians’ right of entry and afterwards failing to provide support. A minority of Australians citizens and residents were abandoned in the government’s pursuit of pandemic border control. This form of abandonment contravenes the moral right to freely enter one’s country, which I will argue should be protected under a more general right to security. Stated differently, the right to enter one’s country is an instance of a security right; being abandoned by one’s government contravenes this right. A somewhat weaker argument would conclude that even if the derogated right of entry was justified, then abandoned Australians deserved more government support than they received. Moreover, the contravening of the right of entry as a security right isn’t just wrong because it harms those Australians who were actually denied entry; rather, it is also a threat to the right of entry as a security right of all Australians in principle. Being secure—in this case, securing the right to enter one’s country—is vital because it allows us as individuals or communities to reliably plan in light of the inherent uncertainties about the future, including pandemics. In turn, such individual- or community-level security is an important way of mitigating risk. At the same time, governments—whether at the federal or state level—have an obligation to protect the health security of the broader public conceived of in totality; however, as I will argue, whatever else is entailed by protecting health security, it cannot come at the cost of some citizens and residents’ right to enter their own country. At the very least, governments who deny their residents entry ought to support them while abroad.

Although perhaps several decisions taken by the current Federal Government in Australia with regards to its borders led to the abandonment or feeling of abandonment of Australian citizens and residents outside the country, I focus on a particular case, namely, the decision in May 2021 to prohibit Australians who were in India to return on penalty of fines or imprisonment. If my argument about abandonment and security is ultimately sound, it would be so at least in this instance. As such, I begin the paper by outlining this case in section two. Next, I argue that Australians in India were abandoned given what is meant by the term ‘abandonment’ itself and because the government did not support those Australians while abroad. In the fourth section of the paper, I will briefly defend the right to enter one’s country. In section five, I will explain what security is and why it is morally important. And in section six, I will argue why I think the right of entering one’s own country should be considered as belonging under the broader right to security and that Australians were unjustifiably denied this right when stranded in India. I will articulate a stronger argument that such a security right ought to be secured absolutely and a weaker argument whereby derogating from a right of entry requires at least supporting those abroad. In sections four and five, I borrow and engage extensively with Jonathan Herington’s important work on the topic of ethics and security.

Before proceeding, some preliminary clarifications: first, when I use the term ‘Australians’ I mean to refer to citizens and residents, since I believe my argument would hold for both and it’s less cumbersome to just simply write ‘Australians’ rather than ‘Australian citizens or residents’. Second, the concept of ‘abandonment’ isn’t always morally problematic, e.g., friends abandon plans, crews abandon sinking ships, etc. The point of my use of the term is not to analyse the moral valence of ‘abandonment’ but simply to say that this instance of abandonment was wrong (I clarify what I mean in section three). Third, as the COVID-19 pandemic has demonstrated, not just in Australia but everywhere, it is difficult and sometimes impossible to untangle which implemented public health measures are endorsed by public health workers as compared to an elected government’s use of purported public health measures that are not endorsed by those working in public health. To the extent possible, I will refer to the Australian Federal Government and their decisions to justify actions on the grounds of the public’s health; I don’t mean to imply that these actions were endorsed by some or a majority of those who work in public health. Fourth, I’m choosing to concentrate my analysis on a very specific case, but I hope that the broader moral argument I’m making will apply beyond this case and be useful to readers outside of Australia. And finally, threatening those Australians in India who wanted to return with imprisonment may be – and likely is – wrong for several overlapping reasons, e.g., it goes against some sense of equality and justice, or was it a racist policy (BBC 2021a), etc. I set aside these other reasons not because I don’t think they’re important, but because they are beyond the scope of this paper.

## The entry ban

On April 27th, 2021, Australian Prime Minister Scott Morrison announced that all flights from India into Australia would cease. On May 3rd, the Federal Government, under the auspices of the *Biosecurity Act 2015*, made it temporarily illegal for Australians who were in India to enter Australia and Australian territories (e.g., Christmas Island), either directly or indirectly (i.e., through a third country) on penalty of fine or imprisonment. The fine was up to $66,000 and the jail term could be up to five years. This prohibition on entering from India lasted for 14 days.

The government’s justification for the ban was as follows: the virulent Delta variant of SARS-CoV2 was being increasingly detected in Australians undergoing hotel quarantine who returned from India (ABC 2021b). Given the increased morbidity and mortality associated with the new strain, officials were concerned that it would escape detection and move from someone in hotel quarantine to the general public in a large, metropolitan city, thus becoming a seeding event for an outbreak. Moreover, there were concerns that the increased stress on the hotel quarantine system would eventually overwhelm it (Dept. of Health 2021). The government understood that the entry ban was “drastic”, but claimed they were acting on recommendations by public health and that it was a necessary step “designed to keep Australians safe” (ABC 2021a). The Prime Minister iterated the need to support Australians in India during the temporary entry ban “over the course of what will be a highly stressful period for those Australians who are caught up or had family members affected by this humanitarian crisis in India” (Martin 2021). Apparently, the Australian government were looking for ways to charter planes to bring back Australians in a safe and controlled manner (Aus. High Commission 2021); however, these attempts were unsuccessful (Minister Foreign Affairs 2021). During that time, the Prime Minister also invoked a solidaristic sentiment, stating that Australians “are standing with them [Australians stranded in India] during what is an incredibly difficult time” (Martin 2021).

What help was afforded Australians who were India, however, remains unclear or were non-existent; it led to calls that Australians were being abandoned and unsupported (Mao 2021). For example, during a question and answer session of a publicly broadcasted news show, one Australian Indian said to the Prime Minister that “…first you grant us exemption to go to India to look after our loved ones who are fighting for their lives, then you abandon us and leave us in a country that is gasping for air (Johnson 2021).” Other Australian Indians were telling their stories on social media, where one person asked: “What nation disowns their own citizens? … All I have left is my mother, who has been abandoned by her own government of Australia, with no way to come back to her children (Visontay 2021).” Politicians from opposition parties claimed that the Indian diaspora in Australia also felt abandoned: “our Indian Australians need our support more than ever before, and the Morrison government are turning their backs on them (Owens 2021).” Another member of the opposition concluded that the issue at hand was “… about all Australian citizens, regardless of their background or where they are at any point in time, being afforded the same respect and support by their own government (Wells 2021).”

It is important to note the context in which this entry ban occurred, namely, that Australia has had throughout the COVID-19 pandemic some of the world’s strictest border controls (BBC 2021b). For example, Australians needed to ask the Federal Government for permission, in the form of exemptions, to enter or leave the country. Tens of thousands of Australians were unable to enter the country throughout the pandemic where there remained “…a persistent sense of abandonment among some expats, who argue the government could have done more to bring more Australians home (Om 2021).” Thus, there are reasons to believe that the sense of abandonment – though perhaps most acute in the case of Australians stranded in India – was felt by many Australians abroad for close to two years.

One might object that feeling abandoned and unsupported does not necessarily mean that Australians in India were actually abandoned and unsupported. Moreover, opposition party members’ articulation of their constituents’ purported sense of abandonment may very well be self-interested. It’s true that feeling unsupported and being unsupported are two different things entirely. And it’s also true that perhaps the government was helping in ways they did not make public (although this seems unlikely given that it would be politically expedient to demonstrate that they were supporting Australians in India despite the entry ban). The question of whether Australians in India were supported by the Australian Government is an empirical question that is not easily answered unless the government clearly articulates what steps were taken to support Australians. Yet given the sentiments expressed by Australians of Indian heritage during that time through traditional and social media *en masse*, there seems to be at least some credence to the claim that they actually were abandoned; it would seem odd to claim that there was some kind of mass delusion about this amongst practically all members of the Australian Indian community.

## Abandonment and support

Australians in India were abandoned, and this abandonment is wrong, in two interconnected ways: first, there is a sense in which being denied entry into one’s own country at all is in-and-of-itself an instance of abandonment. Although stipulative definitions of words are rarely helpful in normative ethics, the etymology of the word ‘abandon’ is illustrative for the purposes of the essay. The morpheme *bandon* appears to date back to Old French and means something like ‘jurisdiction’ or ‘authority’ or under one’s control, so to *a-bandon* means to surrender or let go that which is otherwise under one’s jurisdiction, authority, or control (OED 2021). It seems like this idea is captured when the word ‘abandon’ is used in ordinary language. For example, when friends abandon a plan, they are surrendering their authority in acting on their plan.^1^ Likewise, a crew that abandons a sinking ship seems to be surrendering their jurisdiction or authority over the ship itself. Critically though, it’s the fact that the ship is sinking that justifies the abandonment in the first place; thus, the justification for abandoning *x* is separate from the description of abandoning *x*.

Returning to the case of Australians stranded in India, Australia has the *prima facie* authority or jurisdiction to secure its borders and regulate the entry into the country of its own citizens and residents. When a state institutes entry bans of its residents, it is turning away or rejecting them, i.e., the government forfeits their control, and with it any correlative obligations, to its residents. One could object that the government was trying to secure the health and well-being of those residents who were in the territory of Australia in May 2021 by banning the entry of a minority of Australians abroad; as such, it retained and never rejected its authority to control borders but rather enforced them more stringently. Both descriptions can be true, however, that the government surrendered its authority to protect some Australians abroad in order to uphold its obligations to protect the broader Australian population from an imported deadly virus. Adopting either or both descriptions does not suggest that the entry ban was right or wrong; I think Australia was wrong in doing so, but I still need complete the argument for it, which I (attempt to) do so in sections four through six below.

Second, the abandonment of Australians in India was morally wrong because of the lack of support by government, as noted in the previous section. In April and May 2021, India was not only besieged by the spread of the Delta variant but was also struggling to deal with the volume of COVID-19 cases. The country lacked some critical resources (e.g., oxygen supplies) necessary to care for those who were sickest. Australians in India were unsure they would receive the care they needed should they succumb to the virus. These Australians not only felt abandoned by the travel ban, but that they were unsupported, too. It suggests that whatever might be morally problematic in terms of abandonment and the entry ban, perhaps had Australians been supported it would have lessened the ill effect, and even overcome the wrongness, of the ban itself. Yet, as argued in the previous section, it seems little to no help was forthcoming from the Australian Government.

One way of justifying the Australian Government’s duty to support Australians in India is via the value or principle of reciprocity. Reciprocity denotes “…a moral obligation involving an appropriate response by *B* commensurate with an action by *A*, where *A’s* action aims to contribute to, or bring about, public good, and where the action involves burdens, costs, or risks of harm to *A* (Silva et al. 2016).” Veins and colleagues suggest that reciprocity “…not only requires that individuals should not be overly burdened by measures to protect public health, but also that individuals are *supported* in a way that allows them to fulfil their obligations [my emphasis] (Viens et al. 2009)”. In this case, one can redescribe the need for Australians in India to remain in India as necessary to bring about a greater public good, namely, not importing the Delta variant of SARS-CoV2. Assuming for a moment that it is true that Australians remaining in India actually conferred a public health good,^2^ doing so came at an incredible cost to those persons. Supporting persons who sacrifice their interests to promote the public’s health is necessary to ethically justify asking persons to take on such burdens in the first place.

An objection to claims of support grounded in reciprocity would be that it was nearly impossible to support those Australians stranded in India; the government can only be required to do that which is reasonable given the circumstances. However, potential future harms must be balanced; instances in which we ask people to sacrifice a right or interest without support or compensation are rare and should generally be avoided (Holm 2020). Again, it is in large part an empirical question whether greater harm would have resulted had the entry ban not be instituted than actually prevented. But a second, graver harm would be that by instituting an entry ban, it does away with a right to be secure, which has long-term ill effects on the right of entry in principle for all Australians; this argument is taken up in section six.

In summary, it seems that Australians stranded in India were abandoned by being denied entry; I will attempt to argue over the next three sections that this abandonment was morally wrong because in doing so the government failed in its duty to treat entry as a security right. In addition, Australians in India were also unsupported while being abandoned, which is itself morally troubling, since if one were to argue that it is okay to temporarily deny entry to one’s own residents, it seems reasonable to believe that the government should provide support, lest these Australians be abandoned and wronged twice over.

## Right of entry

There exists plenty of debate in the literature about the nature of rights, their justification, and how they should function. I will (mercifully) not enter this debate. For my purposes, a right delineates an entitlement or claim, *x,* with a correlative duty, *y*. In any particular instance, there’s an identified or identifiable rights-holder and some other identified or identifiable agent that is responsible for fulfilling that right. I believe that the arguments below would apply whether one is a will theorist (i.e., the goal of rights is to give the right-holder control over the duties of another) or interest theorist (i.e., that rights protect and promote a right-holder’s interests). Finally, although I use human rights documents below, I do so to see whether they can provide some normative guidance; I am only interested in moral rights and not legal rights.

The right to enter one’s country is articulated in the *Universal Declaration of Human Rights* (1948); article 13(2) reads: “Everyone has the right to leave any country, including his own, and to return to his country” (UN Gen. Assembly 1948). Moreover, the *International Covenant on Civil and Political Rights* (ICCPR 1966) article 12(d) reads: “No one shall be arbitrarily deprived of the right to enter his own country”. It seems like the justification for this right stems from “the special relationship of a person to [their] country (UN HRC 1999)”. Moreover, other human rights are dependant upon the ability to leave and enter one’s country.^3^ It’s important to note that this right under the ICCPR is derogable under exceptional circumstances, so long as it is not arbitrary (i.e., reasonable given the circumstances and in keeping with the objectives of the ICCPR, namely, to promote the human dignity of all persons). The authors of General Comment no. 27, which provides an official analysis of ICCPR article 12, could not think of any situations that would justify banning the entry of one’s own citizens or residents. In 1963, Ingles – a Special Rapporteur to the United Nations – wrote that the presence of an infectious disease is an insufficient reason to deny entry:

As regards entry into a country, nationals are normally favoured over foreigners and it is unlikely that health or sanitary regulations which prevent the entry of a foreigner would also bar a national. What usually happens is that a national who has an infectious or loathsome disease, for example, is admitted for treatment, or compliance with health or sanitary regulations, instead of being turned away. This arises from the fact that a State cannot shirk its responsibility towards a national or arbitrarily deprive him of the right to enter his own country (Ingles 1963).

Finally, there do not appear to be any instances of persons not being allowed to enter their own country on public health grounds in the human rights literature.

Setting aside the human rights scholarship, there seems to be at least two ways to morally justify a right of entry into one’s own country: first, people live in a highly interconnected world that transcends borders in many facets of life. People simply need to move in-and-out of countries for countless reasons, e.g., for commerce, to take up employment, to see family, etc. Being assured that once you leave your country of residence, you can go back safely and without hinderance becomes imperative. It allows us to leave our country of residence with the peace of mind that we can return home. This reasoning seems defensible through the invocation of interests (i.e., we have an interest in leaving our country of residence and a subsequent interest in returning home) or will (i.e., as a small-scale sovereign, to borrow from HLA Hart, being able to move about, including in and out of countries, seems *prima facie* necessary as a sovereign in modern-day life).

Perhaps a second justification can be found if one considers the very nature of states and borders. If one believes that states with borders are morally permitted or necessary, then it follows that persons entering and exiting would be subject to rules and that states are responsible to set such rules given certain constraints. Although it seems obvious, it bears noting that given the state system we’ve adopted since The Peace of Westphalia, people should belong somewhere since states are entrusted to help protect and promote the well-being of people, namely its residents. Belonging nowhere, or being stateless, deprives a person of many of the goods necessary for well-being or flourishing that are provided by, or protected by, the state. In other words, although perhaps one can reasonably debate whether states should be sovereign in the manner that they exist today, once states do exist, then it becomes imperative for everyone to belong to at least one. If that’s true, then it would seem to follow that physically existing in a place is a right we have if we are to avoid statelessness or placelessness; in turn, we need to be able to exit and return to one’s state of residence.

Regardless of the justification for a right to enter one’s own country, it seems uncontroversial that the state is responsible for regulating its borders, including the rules for entry for its own citizens and residents. However, a constraint on the rules a state may enact regarding how to govern its borders would be that entry should be available to its residents for the aforementioned reasons.

## The moral relevance of security

By ‘security’ I mean that (a) *x* has an interest *y*, and (b) *x* is assured of *y* at some point in the future. I use the concept of interest, but for the purposes of this paper *y* could be conceived of as an interest, a preference, a right, etc. Moreover, *y* can be a good or a process, e.g., I have an interest in being free or I have an interest in the way democratic processes help me to be free. The critical part of security, that separates it from other interests or preferences, is the second clause, where *x* is assured of *y* in the future. In other words, the crux of security is that I can rely upon *y* existing for me for in the future. Herington describes how “…almost all accounts of security … make a claim that what it is for some entity to be secure is to hold a set of conditions or goods *reliably*. At the heart of the structure, its common core, is a shared notion of reliability [emphasis in original] (2012: 15)”. He goes on to note that it is the concept of reliability that “…distinguishes security as a specific, and perhaps especially valuable, moral concept” (Herington 2012: 18). Thus, to capture the important moral valence of security, the second clause of the definition of security at the outset of this paragraph should likely read: (b) *x ought to be assured of y* at some point in the future.

Another complimentary articulation of security, and one that helps flesh out the normativity of the concept, is security as the “inverse of risk or vulnerability” (Wolff 2012). In their book *Disadvantage*, Wolff and de-Shalit note that some of the risks some people face are forms of, or lead to, disadvantage (2007: 68–73). For example, some people will take on employment that poses a risk to their bodily safety that they wouldn’t otherwise accept but for being in poverty, e.g., persons living in poverty in low-income countries working in mines that often lack basic safety measures. As such, one disadvantage (poverty) compounds and leads to more disadvantage (taking on risky employment). Taking on those kinds of risks, then, makes one’s life more insecure. In other words, disadvantage makes the attainment of some future goods unreliable.

The moral thrust of security then is that certain future states of affairs ought to be secure, or held reliably, lest a person be wrongfully disadvantaged. What exactly should be made secure is up for (moral) debate. In abstraction, it does seem at least two contours would shape what goods or processes are secured: first, those things that are foundational for the attainment and enjoyment of other goods would likely be candidates worth securing. For example, many (or perhaps most) ethicists would argue that food and shelter should be secured for all persons, at least some baseline amount or kind. Another example might be the rule of law, i.e., we want to be sure that all persons will be treated the same way by the law. In the case of the rule of law, it’s a process or practice that ought to be secured. Second, we want foundational goods and processes secured because of the inherent uncertainty about the future (what Herington refers to as the robustness of our reliability in a good or process) (2012: 19). Stated differently, we want assurances that some key goods and processes will exist in the future irrespective of future uncertainty. Having these foundational goods secured is imperative for planning, particularly as it relates to planning in light of the future uncertainty that exists. For example, food, shelter, and the rule of law should be secured not only because of their foundational nature, but because being sure those things will exist for us in the future allows us to plan in the first place. As Wolff and de Shalit note, “[t]hose facing uncertainty in employment or housing may find it very difficult to plan for other aspects of their life…” (2007: 69). Thus, the absence of certain goods or processes makes planning about our futures difficult or impossible.

Critically, not everything can be secured, since doing so comes at a high price. As the recent literature on risk imposition makes clear, modernity means taking risks and placing other autonomous persons in the way of risks (cf. Hansson 2013; Oberdiek 2017), e.g., the varied forms of transportation all have risks associated with them for all persons. Indeed, one of the key moral quandaries in policy and research on security is the balance between security and liberty, where ensuring more of one may come at the cost of the other. That is not to say that security isn’t necessary for liberty or autonomy; indeed, to reword what I stated in the paragraph above, securing food and shelter are necessary for persons to plan as autonomous agents. To say that a balance must be struck between security and liberty is not to say that they are always in conflict, just that they may sometimes be in conflict. Anti-terrorism measures, such as increased surveillance through means that invade our privacy, e.g., tracking online activities, is a classic example of this tension. Therefore, the cost of security means that we cannot secure all of our interests or preferences, especially since doing so would likely go against the interests and preferences of others. That which ought to be secured must be critical for persons or communities.

## The right of entry as a security right

Some interests can be treated as entitlements and thus become a right; if these entitlements are to do with security, then they are security rights. It would seem that (a) interests that pertain to foundational goods or processes, (b) are necessary to have reliably for future planning and risk mitigation, and (c) require assistance from others to guarantee that interest’s fruition seem to be likely candidates for security rights. The ultimate guarantor of security rights are states and their governments since they are imbued with the legitimate power of coercing citizens toward cooperative ends that everyone can enjoy as individuals or groups.

The right of citizens and residents to enter one’s own country should be upheld as a security right. As noted in section three, the ability or interest persons have in leaving and entering one’s country can be defended on the basis of the need to travel in modern life, plus the coherence of leaving and entering being part of what it means for states with boarders to exist and for people to belong to a state, i.e., not be stateless as a result of not being allowed back in when they leave. The right to enter one’s own country is a foundational good that requires a correlative process to ensure the obtainment of it as a good and a right, i.e., it requires rules that ensure that leaving and returning are done in an orderly manner. Moreover, knowing that one can enter one’s own country after leaving it would be crucial to all aspects of life planning. The duty to ensure that citizens and residents can use their right of entry would belong to the government given its legitimate use of coercive powers on behalf of the state and its people.^4^

To return to the case of the Australians in India who were denied re-entry in May 2021: not only were they abandoned in the strict and descriptive sense of the term, as outlined in section two, but they were morally wronged in doing so beyond being denied support as necessitated by reciprocity. At the very least, the lack of support they received while stranded in India was another failure by the government that violated a security right they were owed. I will take each of these points in turn.

First, the abandonment of Australians in India was wrong because it transgressed their moral right of entry but moreover, because their right of entry was derogated as a security right. The abandonment was acute in this instance not only because it meant that the plans they made and relied upon were uprooted, but also because they were uprooted when they most needed something to rely on given the uncertainty that surrounds a pandemic. Pandemics and other emergencies are precisely those moments when so much of one’s life is uncertain and subject to unanticipated change. If foundational goods and processes ought to be ensured, it is even more important that they are during emergencies, i.e., security rights, like entry into one’s own country, ought to be robust enough to withstand practically any foreseeable and unforeseeable situation since it’s foundational or basic. Barring the fall of a state itself, the right to move, including the right to exit and entre one’s country of residence should be absolute; too many other goods and plans are grounded on this right being assured absolutely.

One might object that security cannot demand that goods are ensured absolutely. As Herington argues: “…reliability likely comes in degrees, rather than as a binary state. The idea that security is a binary state seems to be recommended by the “secure/insecure” dichotomy, but upon reflection it seems obvious that security must come in degrees. Total security seems impossible… (2012: 18)” To guarantee a right of entry as an absolute, even if it were possible, comes at too high a cost. In the case of Australians in India, the cost of ensuring their return might have been the public’s health. A first response would be that perhaps there are certain goods that could be ensured absolutely, at least in abstraction; negative rights or negative liberty, for example, are at least conceptually easier to secure since they require the duty-holder to not interfere. It might be that the right of entry should be thought of as a negative right and as such, ought not to be interfered with despite being regulated by the state. So there seems to be some sense in which it is possible to secure some rights totally or absolutely.

The second aspect of the objection is more important, namely, that security rights, such as entry, cannot be absolute because sometimes, in some extreme situations, we need to trade it off against another right or good we want secured. Health security would appear to be one such candidate. Much like the concept of ‘security’, the concept of ‘health security’ is equally difficult to define concretely and, as noted by McInnes, the definitions that exist are themselves value-laden (or “promoting a certain agenda and privileging certain interests over others” (2014: 7)). For Herington, “‘health security’ involves distributing security (i.e. cumulative probability) towards initial increments of population health; thereby reducing, *ceteris paribus*, the probability of public health catastrophes (2016: 480).” Herington’s definition captures the desire of security to reduce risk, in this case to population health. One challenge in defining health security has to do with the slipperiness of the concept of ‘health’ in the first place. It seems difficult to speak of securing health, but rather we mean that aspects of health, such as healthcare resources, can perhaps be secured (Wolff 2012). Regardless, the second aspect of the above objection would be that health-related goods (in some sense and capacity) ought to be secured, too, and thus, should be balanced against other things we need secured. Stated differently, health (again, more precisely health care) is the kind of thing that we want secured in the first place given its role in helping people secure other interests and goods, as well as its role in future risk mitigation. Entry into one’s country isn’t the only interest that should be secured, so it requires articulating why it ought to always trump other secured interests, like health.

This objection could be furthered by arguing that health security should be understood as a right to health security, and that this security right should be grounded in a right to public health. Health security could be defined as a right in the form of (a) *x* has an interest *y*, where *x* is a person or population and *y* is health and (b) a person or population is assured, or ought to be assured, of health at some point in the future. It then follows that the normative reasons presented in defence of security apply to health, too. The content of a right to health security could be filled by reference to a right to public health, which must be balanced against securing the right of entry in extreme circumstances like pandemics. The right to public health, as Wilson notes, exists because there are social factors that impact on people’s health (i.e., the social determinants of health) and they are controllable (2016). These factors can be controlled by a government and doing so would greatly improve the health of persons, at least as it relates to removing risk factors that are detrimental to health. Moreover, a government’s ability to shape the social determinants would also improve residents’ chances of exercising their autonomy. Pandemics on the scale of COVID-19 are rare events and pose potentially catastrophic harm to populations if measures are not taken to stop its spread; in other words, governments do have social mechanisms to reduce the risk of transmission associated with COVID-19. The Delta variant of SARS-CoV2, when less was known about it in April and May of 2021, required governments do as much as possible to arrest its spread. In the case of Australia, the right to public health and to public health measures to arrest the spread of Delta might very well have included the obligation to close its borders on some small portion of Australians in order to protect the majority of Australians in the country itself. When two rights cannot both be protected at the same time, and there’s no other principled reason to give preference to one over the other, then protecting the health of the majority could be a sufficient justification to enact a measure that would be detrimental to a very small minority of the population.

The objection, in summary, is that the right of entry cannot be held as a security right absolutely since there’s a competing claim, often in the form of a competing right, to health and public health measures, especially during emergencies like pandemics. Securing the right of entry as an absolute would come at too high a cost, namely, health in this case. Moreover, in situations where health and the right to enter one’s country must be balanced, it seems plausible to side with doing right by the majority, all else being equal. A government cannot always protect everyone at all times, so it is reasonable that they’ll side with the majority’s good in certain extreme instances.

In the actual case of Australians in India, it’s not clear that the federal government could not have upheld its duty to protect the broader public from COVID-19 while allowing Australians to return from India during the April/May outbreak of Delta. Though ultimately an empirical claim I can’t properly defend given my lack of expertise in infection control, I would note that the Delta strain was already global by the time the government instituted an entry ban from India, yet only those Australians in India were banned from entering for two weeks, calling into question the potential futility or arbitrariness of such an entry ban. So assuming that the arguments put forth by Prime Minister Morrison’s government were sound, it is still not clear why it required treating Australians in India differently.^5^

But there’s a second reason why the abandonment of Australians in India is troubling, namely, that any derogation to the right of entry for some makes it unreliable for all Australians in principle. First, the right of entering one’s country can be wholly secured by the state, whereas ensuring the effectiveness of public health measures cannot. One of the reasons we want to secure the right of entry was precisely because it can be assured even during times of great uncertainty. The fact that it can be assured and is a foundational good, seems to be a reason to do so during times of uncertainty when other foundational goods (i.e., health or public health) cannot be assured. Doing away with some category of uncertainty during emergencies, when planning for the future becomes difficult, should be prioritized against those foundational goods we may want to secure but cannot with any certainty. This reasoning applies not just in the particular instance Australians in India, but for all Australians as it relates to entering their own country during the COVID-19 pandemic.

Second, if one person’s right of entry is not secured absolutely, then it signals to everyone that in emergencies, being part of a minority group can lead to being abandoned by your state. No one knows whether at some point in the future they will be part of a (numerically, not socially or politically, speaking) minority group. It’s likely that most people at some point in their lives will be part of a minority. It seems reasonable that people will want a certain set of rights and interests assured absolutely, even if they should happen to be part of a minority group at some point in the future. Given that abandonment in the form of being barred from entering one’s country makes you even temporarily stateless, even the risk of this occurring would severely alter everyone’s behaviour and planning, while also being severely traumatic. If faced with the hardships that come from not being allowed to enter your home country or risks associated with an infectious disease outbreak like COVID-19, it seems the risks of being barred are greater than the risk from infectious diseases. Likewise, seeing other Australians abandoned for the purported-health of the majority should make all Australians question what it means to be a citizen or resident of Australia. In short, some foundational goods should be lexically prior to other foundational goods because many more other goods are built upon it (to extend the metaphor) and as such, should never be sacrificed even at the cost of a minority of individuals.

The above is what I’d call the stronger argument for how the abandonment of Australians in India was a morally inappropriate derogation of a security right; however, there is a second weaker argument. You may still may think that reliability can only ever be a matter of degrees and cannot be assured absolutely, i.e., you find the argument above unpersuasive. Still, if it’s true that the Australian Federal Government did not support Australians stranded in India, this too is a form of abandonment and violation of their security right. I argued in section three that the government had a reciprocal responsibility to support Australians abroad if they were to be denied entry to protect the public’s health in Australia. If it’s true that the right of entry is a security right, then any permitted derogation would be only to the extent necessary to protect the public’s health and no more. A right is still being curtailed and, thus, some responsibility exists to lessen the ill effects of even justified curtailments. This responsibility follows from the claim that reliability is a matter of degree and not a binary; if a good cannot be secured completely, then it should be secured to the extent possible given the circumstances.

In short, the Australian government’s abandonment of Australians in India was wrong on at least one of two grounds, if not both: first, because the abandonment of citizens and residents should not have occurred given the right of entry as a security right should be absolute. But second, and at the very least, if there are exceptions to the reliability of a security right in exceptional circumstances – like pandemics – then the government still had a duty, based on reciprocity, to support those Australians stranded in India.

## Conclusion

The applicability of the preceding argument, assuming it’s sound, is in one important sense universal. There’s no reason to believe that a right of entry as a security right would not apply to all persons regardless of their country of residence. Moreover, it would mean that no pandemic should be used as a grounds for excluding one’s own citizens and residents abroad from coming back home. In another sense, however, the details of a particular situation matter immensely to determine what exactly a government has to do to fulfil its duty to support citizens and residents abroad during a pandemic. *How* the right of entry should be ensured during emergencies is still an important question and one that, I think, would be context dependent; however, *that* a right of entry should be absolutely guaranteed should not.

## Footnotes

1 Their authority to act upon the plan in the first place can be grounded in a myriad of ways (e.g., interests, preferences, etc.) none of which are important for the topic at hand.

2 Although it is beyond the scope of this paper, one might even question the extent to which banning Australians from India conferred a public health benefit. For example, from January to May 2021, less than 1% of all returning travellers tested positive for COVID-19, despite the Delta variant being first identified in November 2020 (NSW Health 2021). It’s unclear just how great of a risk returning travellers from India posed to the health of Australians in the first place.

3 Thank you to Roojin Habibi for drawing my attention to this.

4 Here there may be a distinction made between the standing of a citizen and a non-citizen resident, insofar as residents can have their residency taken away and be removed from the country, e.g., due to being found guilty of a crime. However, here I’m not interested in these situations since such persons are – presumably – citizens of another country. Moreover, there are clear processes, with appeal mechanisms, in place for such situations. There have been morally troubling cases where countries have deported residents to other countries they’ve not known since they were young children (e.g., the Windrush Generation in the UK). Such cases and the details of how to justly deport a non-citizen resident are beyond the scope of this paper.

5 The only other instance during the pandemic where a group of Australians were treated differently from other Australians abroad was when, in late January and early February 2020, Australians returning on charted flights from China were quarantined on Christmas Island prior to being allowed back on the mainland.
